# Auditory Cortical Contrast Enhancing by Global Winner-Take-All Inhibitory Interactions

**DOI:** 10.1371/journal.pone.0001735

**Published:** 2008-03-05

**Authors:** Simone Kurt, Anke Deutscher, John M. Crook, Frank W. Ohl, Eike Budinger, Christoph K. Moeller, Henning Scheich, Holger Schulze

**Affiliations:** 1 Leibniz Institute for Neurobiology, Magdeburg, Germany; 2 University of Ulm, Institute of Neurobiology, Ulm, Germany; 3 Division of Psychology, School of Biology, Newcastle University, Newcastle upon Tyne, United Kingdom; 4 Clinic of Neurology II, Otto-von-Guericke-University Magdeburg, Magdeburg, Germany; 5 Experimental Otorhinolaryngology, University of Erlangen-Nuremberg, Erlangen, Germany; Freie Universitaet Berlin, Germany

## Abstract

Brains decompose the world into discrete objects of perception, thereby facing the problem of how to segregate and selectively address similar objects that are concurrently present in a scene. Theoretical models propose that this could be achieved by neuronal implementations of so-called winner-take-all algorithms where neuronal representations of objects or object features interact in a competitive manner. Here we present evidence for the existence of such a mechanism in an animal species. We present electrophysiological, neuropharmacological and neuroanatomical data which suggest a novel view of the role of GABA_A_-mediated inhibition in primary auditory cortex (AI), where intracortical GABA_A_-mediated inhibition operates on a global scale within a circular map of sound periodicity representation in AI, with functionally inhibitory projections of similar effect from any location throughout the whole map. These interactions could underlie the proposed competitive “winner-take-all” algorithm to support object segregation, e.g., segregation of different speakers in cocktail-party situations.

## Introduction

The parcellation of sensory input into perceptually distinct objects is a basic ability of fundamental importance for all higher animals (e.g. [1]–[3]). However, the neuronal mechanisms by which multiple and often similar objects that are concurrently present in a scene can be separated are presently not understood. It has been suggested (e.g. Ref. [4]) that this can in principle be accomplished by a so-called winner-take-all algorithm. In general, a winner-take all computational algorithm describes a process where several active elements in a (neuronal) network compete for the resources of the whole network, resulting in a state where one element (the “winner”, which for example is the most active element) suppresses the activity of all other elements in the network and thereby remains as the only active element within the network while all other elements are inactive (“losers”). In the context of sensory scene analysis this means that the neuronal representation of one perceptual object suppresses that of other concurrent objects. Despite ample demonstration of the usefulness of the winner-take-all algorithm in theoretical work [5]–[8] physiological evidence for its existence in living brains is still lacking (e.g. Ref. [9]).

**Figure 1:**
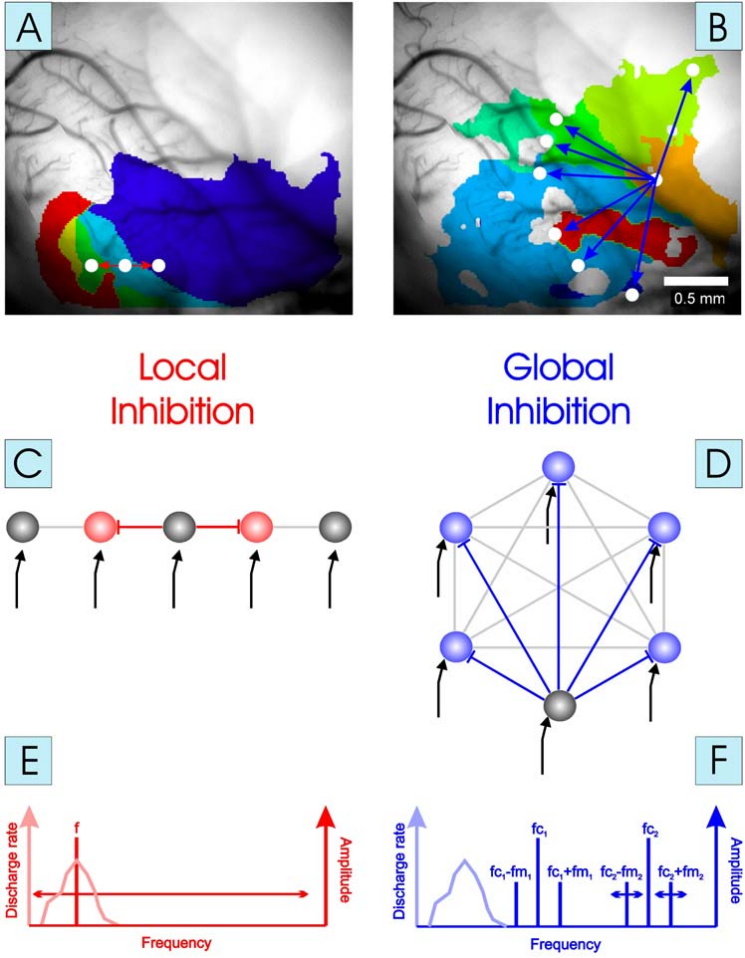
Conceptual framework of the study. The concept of local cortical (lateral) inhibition (left) is contrasted with that of global cortical inhibition (right), as illustrated by examples of a tonotopic and a periodicity map obtained by optical imaging in gerbil AI (A,B, cf. Ref. [17]) and by schematic drawings of the interconnectivity pattern (C,D). A: Different colors within the tonotopic map depict representations of different pure tone frequencies from low (blue) to high (red). B: Different colors within the periodicity map depict representations of different AM tone periodicities from low (blue) to high (red). The concept of local (lateral) inhibition proposes an inhibitory interconnection pattern, whereby any given unit (or stimulus representation) inhibits only its immediate neighbors within the parameter space (A,C: red projections), resulting in local contrast enhancement. The concept of global inhibition proposes an inhibitory interconnection pattern, whereby any given unit (or stimulus representation) inhibits all other representation within the parameter space (B,D: blue projections, inhibitory interneurons are not shown), resulting in the implementation of a “winner-take-all” algorithm, i.e. global contrast enhancement. Note that since any BP representation in a circular map has an eccentric location, projections from any BP representation within the map to all other locations of the map (blue lines) result in an asymmetric geometry of projections (cf. Fig. 6B). Gray lines: inactive projections. Black arrows: Thalamic input. E,F: Stimulation paradigms (schemes) used to test local (red) and global (blue) inhibitory concepts, respectively. E: Isointensity frequency response functions (light red curve) are usually obtained by plotting pure tone (vertical dark red line) evoked discharge rates as a function of tone frequency. F: Stimulation paradigm used here in the competitive interaction experiment. Two AM tone complexes were presented simultaneously (duration: 200 ms, 65 dB SPL). Spectra of both AM tone complexes (vertical dark blue lines) were entirely outside the frequency receptive field (FRF; light blue curve; cf. Ref. [33]) of the unit, which in our experiments always meant above the FRF, because all recorded units showed responses to low frequency pure tones. One of the complexes had a fixed fm (fc1/fm1) set to best periodicity of one of the units, the second had a different fc (fc2) and varied in fm (fm2). Note that fc2 could be higher or lower than fc1 in the experiments, but both AM spectra were always completely above the units' FRF.

In central sensory systems perceptual objects are believed to be formed by binding together stimulus features that belong to the same object [10]. Such features are represented in functional maps, in which the parameter space of a feature is systematically analyzed by neuronal filters each selective for a certain range of the parameter space. Therefore, the physiological implementation of a winner-take-all algorithm poses specific constraints on the functional organization of neuronal interconnectivity patterns and their recruitment during stimulus processing. Here, we hypothesize that a winner-take-all process would require a neuronal interconnectivity pattern by which any location within a feature map is allowed to inhibit all other locations in a global fashion (cf. Fig. 1B,D). In the case of multiple sensory objects which differ in the feature that is represented within the map this would lead to a thalamic input to multiple areas within the cortical map resulting in an initial state with multiple active spots within the map. These active spots would then activate inhibitory interconnections between each other, and the strongest inhibitory input should be provided by the most active spot within the map. Consequently this spot might receive less inhibition than it would impose on other locations, resulting in an activity pattern within the feature map where only one spot which had the strongest activity in the beginning (the “winner”) would still be active while all other locations in the map would be silent. Note, that this slightly higher activity of the winner not necessarily has to result from stronger thalamic input but could as well be the result of some top-down influence of higher cortical areas, e.g. of those that control attentional demands [11], [12].

It is notable that in contrast to this idea, intracortical inhibition in AI has so far been conceptualized predominately on a local scale, i.e. as lateral inhibition, whereby neurons in a given location of a map inhibit only their direct neighbors with a decline of inhibitory influence according to some space function (cf. Fig. 1A,C; e.g. Refs. [13]–[16]), and long-range inhibitory projections have only very rarely been demonstrated [17].

For the auditory cortex, local inhibition is supported by studies demonstrating that a stimulus within an inhibitory sideband of a neuron's receptive field can suppress responses to a stimulus concurrently presented within the excitatory center [18]–[20], or that excitation and inhibition of neurons are co-tuned, i.e. they show approximately the same dependence on frequency and intensity of a pure tone stimulus [21]. Also neurons exhibiting multiple inhibitory areas can be considered as reflecting local inhibitory influences [22]. For these types of inhibitory action it is not clear, however, whether they occur in cortex or are transmitted from some subcortical level (cf. Ref. [23]). Microiontophoretic studies in auditory cortex with pharmacological blockade of GABA_A_-mediated inhibition can provide more direct evidence and have shown broadening of frequency tuning curves with bicuculline (BIC) [13]–[16]. But this effect is controversial as it is not seen with the more specific GABA_A_-antagonist gabazine [23] pointing to the possibility that at least part of the extensive GABAergic neuronal systems in auditory cortex serve other than local inhibitory functions. Based on these contradictory data, the whole concept of sensory neurons acting as feature detectors is currently under debate, and it has been suggested that neuronal activity within auditory cortical maps represents auditory objects rather than stimulus features [24], [25].

A suitable substrate to test for more global mechanisms of inhibition under the winner-take-all concept is a recently described periodicity map in gerbil auditory cortex [4]. This map is functionally superimposed on the tonotopic map of the primary field AI. The almost circular functional gradient for different sound periodicities is a geometry that could support inhibitory connections of similar effect from any location throughout the map. An interesting applied aspect of competition between different sound periodicities is its implication for voice segregation in a cocktail-party situation [26].

Here we have used a combination of electrophysiological, neuropharmacological and neuroanatomical techniques to investigate whether a global inhibitory interconnectivity pattern for object segregation is indeed realized in the circular periodicity map of the auditory cortex. We specifically address the following questions: (1) Does the neuronal activity which represents a certain stimulus within the circular periodicity map suppress the neuronal response to a concurrently presented second stimulus which is represented somewhere else in the map? (2) If so, is this suppression mediated by GABA_A_-mediated inhibition? (3) Do direct projections within the map have the appropriate length and topography to interconnect different frequency domains of the cyclic periodicity map; and if so, do these projections terminate on inhibitory interneurons in order to provide an anatomical substrate for suppressive competitive interactions between representations of different periodicities?

## Results

### Simultaneous recordings demonstrate competitive interactions within AI

In a first set of experiments we tested the hypothesis that inhibition within AI acts globally, implementing a “winner-take-all” algorithm for sound object segregation on the basis of periodicity discrimination. In 6 anaesthetized animals, we made simultaneous recordings in two regions of AI representing different periodicity ranges. Recordings were made in situations where only one amplitude modulated (AM) tone (i.e. one auditory object with a particular periodicity) was presented as well as in situations where two AM tones were presented concurrently (see Fig. 1F). Responses from a total of 24 simultaneously recorded pairs of units were obtained. All these units were located in the low frequency area of AI and had BFs ≤5 kHz. Of these, 19 showed a response behavior consistent with the global inhibition hypothesis.

**Figure 2:**
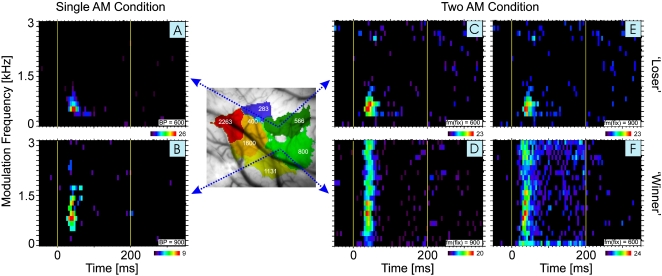
Competitive interactions in a cell pair recorded simultaneously in AI. Response plane histograms are shown for 2 units (A,C,E and B,D,F) with different best periodicities (600 and 900 Hz, respectively) and hence located in different areas of the circular periodicity map in AI (as schematically indicated by arrows from the periodicity map between left and middle column; numbers in map refer to BPs of the respective map area) which were recorded simultaneously during stimulation with either a single AM tone complex (A,B) or 2 concurrently presented AM tone complexes (C–F). Response plane histograms are arranged in panels where modulation frequency of AM tone complexes (in steps of 150 Hz) is plotted over time. Spikes per bin are color-coded according to the scale bar at the bottom right of each histogram, with the maximum number of spikes indicated in each case. Histogram binwidth: 5 ms. Vertical yellow lines mark onset and offset of stimulation. In the 2 AM tone condition in C,D, the best AM tone complex for each unit was fixed, while the concurrent AM tone complex was varied in fm (cf. Fig. 1F). In E,F the fixed AM tone complex was that of the other unit, that is, C,F and D,E (and A,B) were recorded simultaneously. The unit in A,C,E (the ‘loser’) responded only to the two AM tone complexes when the periodicities of both AM tone complexes fell within its periodicity receptive field. By contrast, the unit in B,D,F (the ‘winner’) always responded to its best AM tone complex irrespective of the periodicity of the concurrent AM tone complex. Note that the strength of the response to the best AM tone complex could be modulated by the second AM tone complex: Strongest responses were typically seen, when both AM tone complexes had similar periodicities, that is, when both periodicities were within the receptive field of the unit.

An example of this type of behavior is shown in Fig. 2 which compares the responses of two units with different best periodicities (BP) for AM tones (A,B), which were recorded simultaneously from neighboring periodicity representations in AI (cf. scheme of periodicity map between left and middle column). The left column (A,B) shows the different responses of the units to AM tones with a common carrier frequency (fc) of 12 kHz and modulation frequencies (fm) which varied between 0 (unmodulated carrier) and 3 kHz. The rate modulation transfer functions of these responses (rMTF = plot of evoked spike rate as a function of the fm of the AM tone) revealed a BP of 600 Hz for the unit in A and a BP of 900 Hz for the unit in B. Similar recordings during stimulation with an AM carrier of 8 kHz revealed identical BP values in both units (not shown).

The middle column (C,D) shows the responses of the same units during stimulation with two simultaneously presented AM tones. In panel C the unit from (A) is always stimulated with its optimal AM tone (fc = 12 kHz; fm = 600 Hz) and in addition with an AM tone of 8 kHz fc and varying fm. This varying fm of the second AM tone is plotted on the ordinate in panels C to F. As can be seen from the response plane diagram, this unit responded only when the fm of both AM tones matched its AM tone receptive field, although its optimal AM tone was present throughout all stimulus conditions. The presence of the second AM tone therefore suppressed the response of this unit to its optimal AM tone as long as the periodicity of the second AM tone fell outside the AM tone receptive field of the unit. In contrast, the second unit (panel D) showed the reverse response behavior. As for the previous unit, the optimal AM tone complex was presented in all stimulus conditions (fc = 8 kHz, fm = 900 Hz) in addition to a second AM tone with a fc of 12 kHz and varying fm (cf. Fig. 1F). In this case, however, the unit responded to its optimal AM tone irrespective of the periodicity of the second AM tone. Note, that this type of response behavior does not reflect a loss of stimulus selectivity of this unit: This unit still responded highly selective to the AM tone with its optimal fm ( = BP), simply “ignoring” other stimuli which were presented simultaneously.

The right column (E,F) shows the responses of both units that where recorded when the fm of the fixed AM tone complex was set to a value different from the BP of the units. As can be seen, the responses look qualitatively similar in E,F as in C,D, although the responses were a little weaker in E,F compared to C,D, respectively. Based on our model of competitive “winner-take-all” interactions in AI this is exactly what would be expected: In the cases where the fixed AM tone had a non-optimal fm, the fm was still in the AM tone receptive field of the unit (cf. A,B), that is, should elicit a weaker response than an AM tone at BP. In combination with the second AM tone, the loser-type unit (E) still responded only in cases, where both AM tones had periodicities in the AM tone receptive field of the unit, that is, under conditions where there was no competitive interaction in AI. Note, that in this experiment the second AM tone with varying fm sometimes had a fm at or close to BP. In contrast, the winner-type unit (F) always responded to the fixed (off-BP but within AM tone receptive field) AM tone, but the response was weaker that in D, except in those cases where the second AM tone hit the BP of the unit or was close to it (within the AM tone receptive field).

The response behavior of these two units seems to be a prerequisite for but not yet evidence of a competitive “winner-take-all” system within the periodicity map in AI. For any given competitive interaction between AM tones there could be units in any BP range of the map whose responses to its optimal AM tone can be suppressed as long as the competing AM are different (losers) and other units that maintain their response to an optimal AM irrespective of the presence of other competing AM tones (winners). Note that the response of the unit shown in Fig. 2A (the loser) is actually much stronger than the response of the unit in Fig. 2B (max bin 26 spikes vs. 9 spikes). Clearly, each stimulus periodicity will activate numerous units within the periodicity map, and our model is that *areas* within the map with units that represent similar periodicities and that are activated by the same stimulus competitively interact with each other. The result of the winner-take-all competition is that the *area*, not any given unit, with the strongest activation will win the competition.

Looking at the ‘loser’-type response in Fig. 2C one might argue that this type of response behavior is not due to the suppressive interaction mechanism proposed by our model but rather reflects adaptation to the best stimulus which was present in all stimulus conditions. To exclude this possibility we performed control experiments (not shown) where a single AM tone at BP was presented 200 times while the second AM tone was omitted. In the five units tested in this manner we did not observe any signs of adaptation.

Of 24 cell pairs tested with this paradigm, 10 showed the above type of behavior, with one unit behaving like a ‘loser’ and the other unit like a ‘winner’. However, in nine pairs both units behaved like ‘losers’. This is qualitatively what one might expect given that we were only able to record from two units simultaneously. If our hypothesis is correct, at any given time there should be many ‘losers’ within the map but only one ‘winning’ BP-representation. For the remaining 5 pairs both units behaved like ‘winners’ (see Discussion). It is also worth pointing out that two of the ten winner-loser-pairs recorded showed a ‘switching’ type of behavior, whereby one unit was the ‘winner’ during the measurements with the first set of two AM tone complexes (where the fm of the fixed AM tone was set to BP of the first unit), whereas the other unit was the ‘winner’ during measurements with the second set of stimuli (where the fm of the fixed AM tone was set to BP of the second unit). This change of a winner- to a loser-type behavior was also observed in five additional units that were recorded separately in single-electrode recordings. This switching behavior might be counterintuitive at first sight, but it has to be expected from a mechanism that is able to dynamically select and segregate an auditory object out of a combination of concurrent objects as the relevant object that has to be attended by a subject may vary over time. We will discuss this phenomenon in more detail below (Discussion section).

### Quantitative comparison of winner- and loser-responses

**Figure 3:**
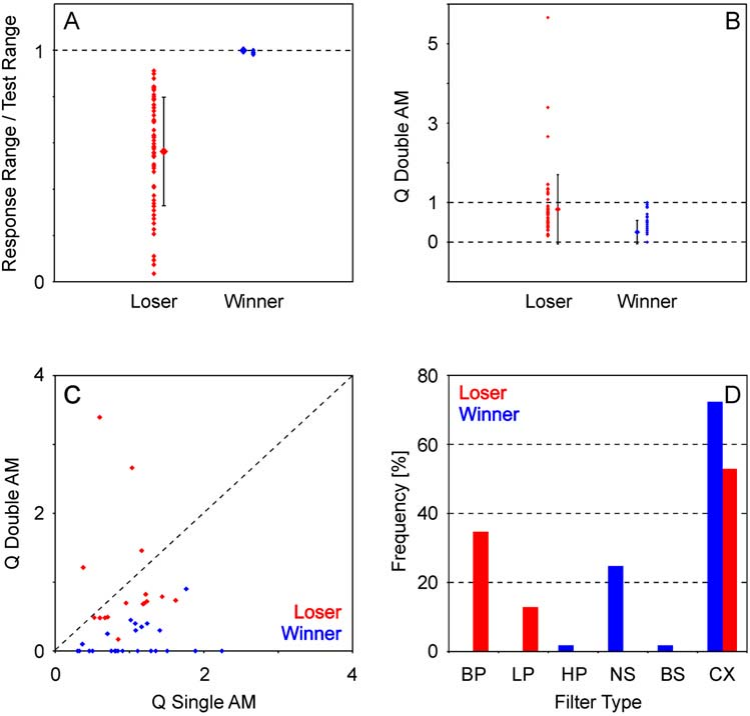
Quantitative analysis of tuning properties of winner and loser responses. (A) Relationship between response range and range of presented stimuli. By definition, winner units respond to all stimulus combinations in two AM conditions. (B) Distribution of tuning sharpness (Q) during two AM conditions. Tuning of losers is significantly sharper than tuning of winners. Depending on BP, Q-values of winners may only range between 0 and 1, whereas those of losers may exceed 1. (C) Comparison of tuning sharpness (Q) between one and two AM conditions. Whereas there is no significant change in Q in losers, winners show significantly smaller Q-values during the two AM condition compared to the one AM condition. (D) Frequency distribution of tuning filter characteristics. Complex (CX) filter characteristics were most frequent in both winners and losers, but whereas only winners showed high-pass (HP), non-selective (NS) and band-suppression (BS) filter characteristics, band-pass (BP) and low-pass (LP) filter characteristics were only found in loser responses.

As described above, a unit was considered a ‘winner’ when it responded to its optimal AM tone complex independent of the second tone complex presented in a certain experiment. A criterion for this decision was a significant excitatory response to all AM tone complex combinations in response plane histograms (cf. Methods). All unit responses that did not fulfill this criterion were considered ‘losers’. After this qualitative classification of responses we performed a quantitative comparison of winner- and loser-type responses on the basis of tuning properties or rate functions (Fig. 3).

Fig. 3A replicates the classification of response type on the basis of rate functions: Here, the response range of the units divided by the range of AM tone complex combinations presented is given for all units classified as either winner or loser. As expected, this value is 1 for all winners (blue), except in three units where the response to one AM tone complex combination dropped below significance although there was a significant response in the response plane histogram (an effect which is due to the different time windows which were used for the two types of analysis). In contrast, the values for the losers (red) vary over a wide range between 0 and 1. Mean values and standard deviations are given between the single data points (Winners: mean = 0.999, SD = 0.003; Losers: mean = 0.563, SD = 0.234).

Fig. 3B compares the sharpness of tuning as expressed by Q-values (cf. Methods) of winners (blue) and losers (red). Although winners responded to each pair of AM tone complexes (per definition) response strength could vary so that filter characteristics and BP could be defined for winner-type responses (the criterion for classification of filter type was set to 50% of maximal response, cf. Methods).

For winners, Q-values were restricted to values between 0 (non-selective filter type where no BP could be determined) and 1 (BP equals upper border of BP presented). In contrast, Q-values of losers were never 0, varied over a wider range exceeding 1 and were significantly larger than those of winners (Winners: mean Q = 0.25, SD = 0.30; Losers: mean Q = 0.83, SD = 0.87; ANOVA P = 3.8^−6^).

A comparison of tuning characteristics of the condition where only a single AM tone complex was presented with the condition, where two concurrent AM tone complexes were presented reveals another difference between winners and losers (Fig. 3C): Whereas the tuning for the winners was always sharper during the single-AM condition compared to the double-AM condition, that is, Q-values decreased from the single-AM to the double-AM condition, Q-values of losers could either decrease or increase from the single-AM to the double-AM condition. A paired t-test revealed that these changes in Q-value were significant across the population of winners, but not significant across the losers (Winners: single-AM: mean Q = 1.04, SD = 0.50; double-AM: mean Q = 0.14, SD = 0.23, paired t-test: P = 2.6^−9^; Losers: single-AM: mean Q = 0.96, SD = 0.36; double-AM: mean Q = 1.00, SD = 0.86, paired t-test: P = 0.44).

Finally, winners and losers showed largely different frequency distributions of filter types of rate functions recorded in response to two AM tone complexes (Fig. 3D): Whereas complex filter characteristics were found for both winners and losers, band-pass and low-pass filter characteristics were encountered only in loser-type responses. High-pass, non-selective and band suppression filter characteristics were found only in winners. Note the high percentage of band-pass tunings in losers and the high percentage of non-selective tunings in winners.

### Iontophoretic application of BIC modulates competitive interactions within AI

**Figure 4:**
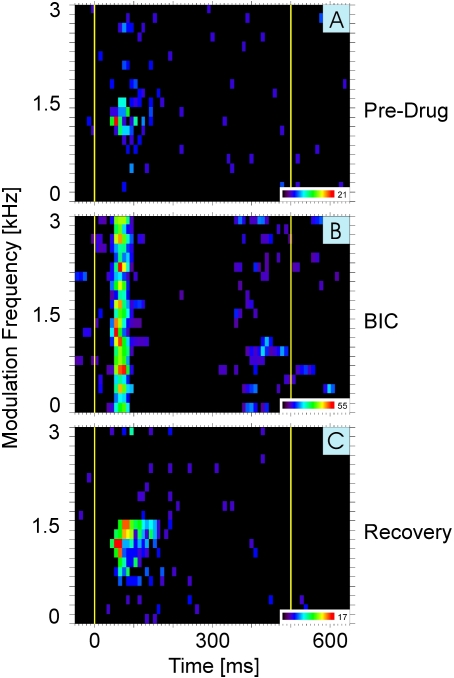
Pharmacological manipulation of cortical competitive interaction. Responses plane histograms in response to two concurrently presented AM tone complexes are shown for the same unit in AI before (A), during (B), and after (C) iontophoretic application of BIC; ejecting current: 40nA. Conventions and layout as in Fig. 2C,D. The unit showed a ‘loser’-like response behavior in the pre-drug condition, but a ‘winner’-like response behavior during blockade of GABA_A_-mediated inhibition via iontophoresis of BIC.

The experiments described above demonstrate the existence of suppression of neuronal representations of some periodicities within the periodicity map in AI during simultaneous stimulation with two concurrent periodic sounds. To test whether the suppressive interactions resulted from GABA_A_-mediated inhibitory processes, we examined the effect of iontophoretic application of BIC on the responses of single AI units to stimulation with two simultaneously presented AM tone complexes in a total of 27 units. We hypothesized that if the suppression were GABA_A_-mediated, a cell showing a ‘loser’-like response behavior might show a ‘winner’-like response behavior during blockade of GABA_A_-receptors via iontophoresis of BIC at the recording site (see Method section). As illustrated in Fig. 4, we found that this was indeed the case.

Fig. 4A shows the response of a ‘loser’ in a stimulation situation with two concurrent AM tones. Similar to Fig. 2C, this unit responded to the combination of AM tones only if the periodicities of both sounds fell within its AM tone receptive field. The response to the AM tone with the BP of that unit (which was present in all stimulus combinations presented) was inhibited by the presence of any AM tone whose periodicity fell outside the unit's AM tone receptive field. Fig. 4B shows the responses of the same unit to the same stimuli during iontophoretic application of BIC. The unit now responded to the AM tone with its BP irrespective of the periodicity of the second AM tone complex. This is indicative of a ‘winner’-like response behavior. The effect was reversible, with the unit again showing a ‘loser’-like response behavior to concurrent AM tones within 20 min of the termination of BIC application (Fig. 4C).

**Figure 5:**
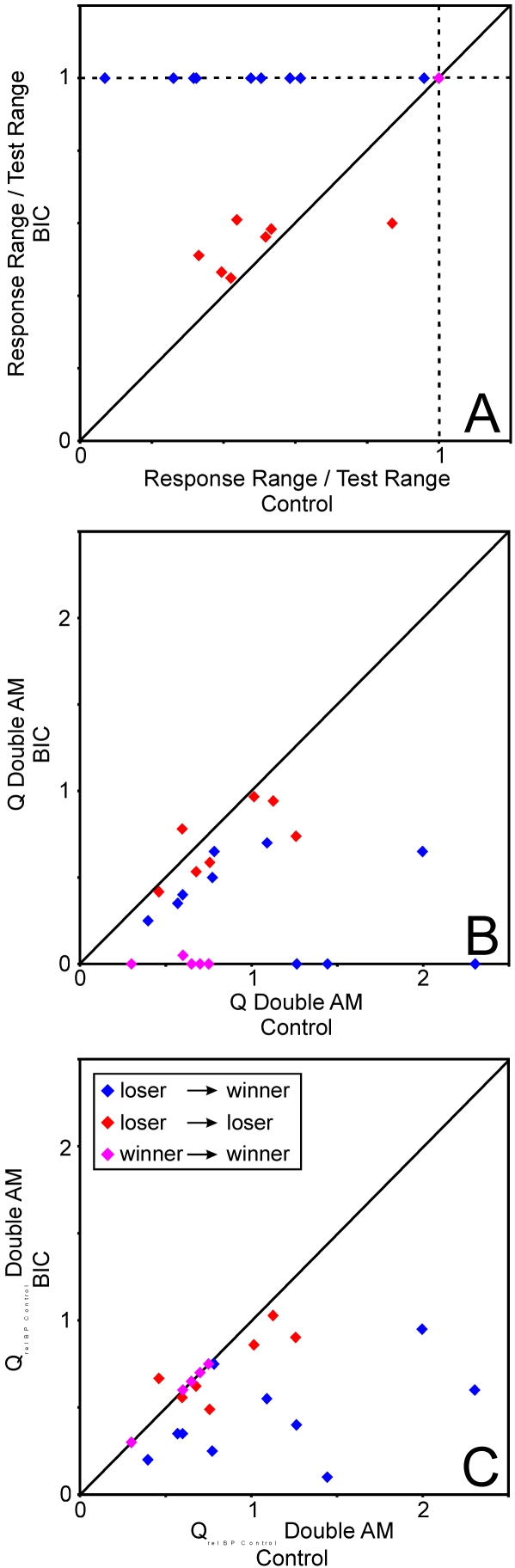
Effect of blocking of GABA_A_-mediated inhibition by BIC on winner and loser responses in two AM conditions. (A) Relationship between response range and range of presented stimuli whereas some units (blue) changed their response properties from loser to winner type, others stayed losers (red) but even then typically responded to a wider range of stimulus combinations (red dots above diagonal). Winners (pink) always stayed winners under drug conditions. (B) BIC-effect on tuning sharpness. Q-values became significantly smaller in those units that changed from loser to winner response type (blue), but this effect was only a trend in the group of losers that stayed losers (red). Winners (pink) also showed significantly smaller Q-values during the BIC-condition because of a drop of BP. (C) A similar effect was seen with a modified Q-value where the BP of the control condition was also used to calculate the Q-value of the BIC-condition. With this analysis consequently tuning sharpness of winners did not change at all (pink).

This type of behavior illustrated in Fig. 4 was observed in 10 of 22 units (the responses of 5 units were to weak to be analyzed quantitatively), (cf. Fig. 5A, blue). Another 6 units showed a widening of the periodicity range under BIC (cf. Fig. 5A, red, dots above diagonal). In the context of our model proposed here this would imply that the inhibition imposed on these 6 ‘losers’ by units activated by the second AM tone could not be blocked completely, but nevertheless – and in contrast to control conditions - they maintained responses to their BP when the competing AM tone had fm in the vicinity of their AM receptive fields. One unit showed a shrinking of the periodicity range under BIC (cf. Fig. 5A, red, dot below diagonal). The remaining 5 units showed a winner-type response behavior before BIC-application and maintained this behavior during BIC-application (cf. Fig. 5A, pink). In summary, all units manipulated with BIC except for the one loser that showed a shrinking of the periodicity range under BIC ( = 21 out of 22) showed response behavior consistent with our model of competitive winner-take-all interactions.

Fig. 5B shows a quantitative analysis of the described effect of BIC on tuning properties of the responses to competing AM tone complexes in AI units: For all units except one the Q-value decreases during application of BIC, but the effect is stronger in units that changed their response behavior from a loser- to a winner-type behavior (blue) than in those that remained losers during the BIC condition (red) (ANOVA: Changes in Q from control to BIC condition in blue vs. red group: P = 0.05). The change in Q from control to drug condition was significant in the blue group (loser to winner, mean Q control = 1.12, SD = 0.63; mean Q BIC = 0.35, SD = 0.28, paired t-test: P = 0.005), but there was only a trend to smaller Q-values in the red group (loser to loser, mean Q control = 0.84, SD = 0.30; mean Q BIC = 0.71, SD = 0.21, paired t-test: P = 0.08). The pink group (winner to winner) also showed significantly smaller Q-values under the drug condition which is, as response range is unchanged (cf. Fig. 5A) due to smaller BP values under the drug condition (mean Q control = 0.6, SD = 0.18; mean Q BIC = 0.01, SD = 0.02, paired t-test: P = 8.9E^−4^).

Because of this dependence of the Q value from a potential change in BP, we performed the same analysis with a modified Q-value, where the response range under both conditions was set in relation to the BP of the control condition (Fig. 5C). This analysis led to similar results: Q_rel BP control_ – values again changed significantly stronger in the blue compared to the red group (ANOVA: P = 0.02). The change in Q_rel BP control_ from control to drug condition was significant in the blue group (loser to winner, mean Q_rel BP control_ control = 1.12, SD = 0.63; mean Q_rel BP control_ BIC = 0.45, SD = 0.26, paired t-test: P = 0.002), but there was only a trend to smaller Q_rel BP control_ -values in the red group (loser to loser, mean Q_rel BP control_ control = 0.84, SD = 0.30; mean Q_rel BP control_ BIC = 0.73, SD = 0.20, paired t-test: P = 0.08). The pink group (winner to winner) consequently showed no change in this analysis, as response range is unchanged in this group.

Finally, to exclude the possibility that the change of a loser-type response behavior to a winner-type response is not induced by the GABA_A_-blocking effect of BIC but rather by secondary effects of BIC [23] we performed control experiments where we repeated the experiment presented in Fig. 4 in three units (not shown) with the GABA_A_-antagonist Gabazine which is known not to have these side effects. In all cases, a change from a loser-type to a winner-type response behavior was observed.

### Neuroanatomical support for global competitive interactions in AI

To investigate whether intrinsic connections in AI are capable of mediating the type of global competitive interactions proposed here, we performed neuroanatomical experiments which combined anterograde tract tracing with immunohistochemical staining of GABAergic neurons. The central issue here was that the BIC experiments showed that a given fm sensitive neuron can be inhibited by all other BP representations but not that a given neuron can inhibit all other BP representations.

To characterize neuronal interconnectivity within AI, we made microinjections of the anterograde tracer biocytin into AI and analyzed the distribution of labeled axons and their terminal boutons. To identify putative inhibitory target neurons, we stained cells against the calcium-binding protein parvalbumin (PV), which labels approximately 80% of GABAergic cortical neurons [27].

**Figure 6:**
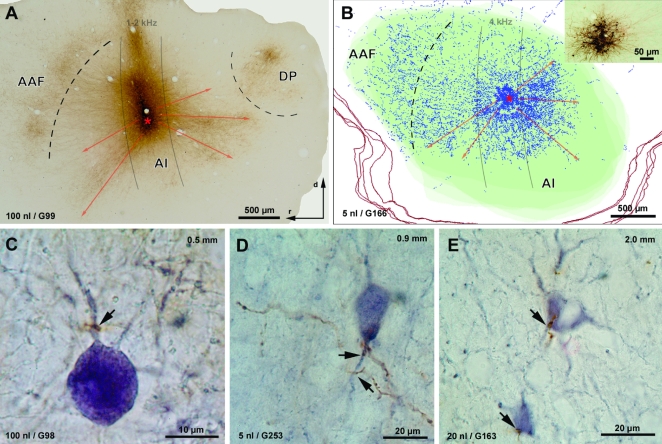
Neuroanatomical support for global, long-range inhibition in AI. Panels A and B show axonal fibers and terminal boutons labeled anterogradely following injections of biocytin into AI of the gerbil. Panels C–E demonstrate direct contacts of biocytin-labeled terminations with parvalbumin-positive (i.e. GABAergic) interneurons. A: Single sagittal section through the auditory cortex processed for biocytin following an injection of biocytin (100 nl) into layers II–IV of AI. Labeled axons (stained brownish-yellow) expand from the injection site (red star, BF 1–2 kHz) to a large extent in dorsal direction, i.e. in a tonotopic manner within the dorsoventrally oriented 1–2 kHz isofrequency contour (schematically indicated by grey solid lines). Additionally, a substantial number of axon projections cross different frequency domains (i.e. are non-tonotopic), in this case predominately in ventrorostral and ventrocaudal directions (red arrows). Note, that these non-tonotopic, long-range projections spread asymmetrically from the injection site (indicated by different length of red arrows). Additionally, projections to the anterior and posterior auditory fields (AAF and DP) can be seen. B: Reconstruction of five consecutive sagittal sections through supragranular and granular layers of the gerbil's auditory cortex following an injection of biocytin (5 nl) into layers III–IV of AI (red star; BF 4 kHz). Red lines represent outlines of traced sections, green-shaded area corresponds to the auditory koniocortex (which comprises fields AI and AAF [14], [28]). Blue stars represent biocytin-filled neurons at the injection site (shown on microphotograph in the inset). Blue dots represent biocytin-labeled boutons. Note again the asymmetric distribution of labeled boutons of the non-tonotopic, long-range projections within AI (red arrows). C–E: Biocytin-labeled terminations (brownish stain) in contact with various PV-positive GABAergic interneurons (violet stain) in layers III/IV of AI. Arrows point to synaptic contacts at proximal dendritic aspects (C, D) and at cell somata (E). The distance of contacted PV-positive somata from the injection site is noted in the top right-hand corner of each panel. Section orientation in A applies to all panels. The experimental cases from which data are shown as well as the volume of biocytin injected are noted in the bottom left-hand corner of each panel.

Three different injection volumes were used for the application of biocytin into AI of 10 animals: 100 nl, 20 nl and 5 nl. These injections labeled 1170±268, 380±78 and 137±47 neurons respectively at the injection site (Table 1). Labeled cell bodies were usually all of the pyramidal cell type. The labeled axons and terminations of these pyramidal neurons were mainly distributed within a slab extending in dorsoventral direction across AI, i.e. in a tonotopic fashion parallel to the isofrequency contours (indicated by grey lines in Fig. 6A, B), and encompassed all cortical layers. This connectivity between neurons of an isofrequency slab within AI as well as between AI and other auditory fields (see also Fig. 6A, B) has previously been described [28]. However, particularly in supragranular layers labeled axons and terminations were also distributed non-tonotopically across different frequency domains (Fig. 6A, B). These long-range projections which extended up to 3 millimeters, characteristically spread asymmetrically from the injection site (indicated by different length of red arrows in Fig. 6A, B). Notably, this asymmetric connectivity pattern might be expected for a projection that originates eccentrically in a circular map and then covers the whole map (cf. Fig. 1D).

**Table 1:** Parameters of biocytin injections.

Animal number	Biocytin [nl]	Diameter of injection site±SD [µm]	Layers covered by injection site	Labeled cells at injection site	BF at injection site [kHz]
G98	100	280±30	II/III-Va	1360	0.5–1.0*
G99	100	260±40	II-IV	981	1.0–2.0*
G163	20	190±30	III-Va	436	2.0
G165	20	180±30	III-Va	325	1.0
G166	5	140±30	III-IV	138	4.0
G171	5	130±30	IV-Va	189	2.0
G172	5	130±20	IV-Va	197	8.0
G251	5	120±30	III-IV	103	5.0–8.0*
G252	5	140±20	III-IV	106	2.0–4.0*
G253	5	110±30	IV-Va	87	5.0–8.0*

Animal number, injection volume, diameter and laminar location of injection sites, number of labeled cell bodies and best frequency (BF) at each injection site are listed for each experimental animal. The diameter of each injection site was measured directly in each of the sections in which it was contained, and the mean diameter was calculated. BFs were determined either electrophysiologically or, in cases of stereotaxically guided injections (*), estimated by the locations of the injection sites relative to external and internal landmarks (see Materials and Methods).

Not only after large injections, but also following very small injections of biocytin (5 nl; injection site diameter 130±10 µm, 137±47 neurons labeled, cf. Tab 1), did we observe a similar axonal projection pattern, indicating that even individual neurons or small neuronal populations have that long-range and asymmetric projections across the tonotopic gradient. For example, approximately 400 of the 25.000 labeled boutons in Fig. 6B were located more than 1.8 mm away from the injection site.

The antibody against PV particularly stained somata and proximal aspects of dendrites of mainly non-pyramidal cells (as well as of few pyramidal neurons in layer VI and various non-classifiable punctae of the neuropil). All layers contained PV-positive neuronal elements, but staining was most intensive in layers III/IV. As illustrated in Figs. 6C–E, biocytin-labeled axons often terminated on various PV-positive interneurons in all layers, but particularly in the supragranular and granular layers. The labeled contacts were made both close to (Fig. 6C) and distant from (Figs. 6C, D) the injection sites.

Taken together, these results suggest that the long-range excitatory projection of a pyramidal cell contacts a distant GABAergic interneuron which in turn inhibits the surrounding pyramidal cells. This interconnectivity pattern could form the anatomical substrate for the suppressive interactions we observed between representations of different periodicity ranges within AI (Fig. 2). The geometry of the described projections shows that connectivities emanating from a given point in AI asymmetrically cover large parts of the map and thereby provide indirect evidence that a given neuron or neuron ensemble can inhibit other ensembles throughout the map.

## Discussion

In this study, we have presented electrophysiological, neuropharmacological and neuroanatomical evidence that GABA_A_-mediated inhibitory processes in AI mediate global suppressive interactions between representations of different AM-tone periodicities. These interactions may underlie a competitive “winner-take-all” algorithm which supports object segregation. That is, in our model the stimulus feature of sound periodicity is used to segregate sound objects that differ in their periodicity. Such a mechanism might be particularly useful in the so-called ‘cocktail-party phenomenon’, where voices of different concurrent speakers can selectively be attended to. Based on our model and consistent with the data presented in Fig. 2 (two AM paradigm), the segregation of two sounds should be easy when the periodicities of the sounds are considerably different (e.g. listening to a man talking while many children are talking simultaneously), but it should be very hard or even impossible if the two periodicities are very similar (e.g. listening to a particular child in a group of children) because here the responses of the “losers” in the winner-take-all interaction are not inhibited (cf. Fig. 2C,E). In this latter case, additional sound cues like sound source location in space will be crucially important for sound segregation (i.e. speaker separation) and the mechanism discussed here would be rather ineffective. But in all cases where sound objects differ in their periodicity – which is the typical situation for voices of different speakers – the winner-take-all mechanisms proposed in our model would be very powerful in speaker ( = sound object) segregation. So based on our model it is not the sound feature (periodicity) *per se* what matters in auditory cortical processing, but what the cortex does with it, namely object segregation of periodic sounds.

The results presented are consistent with our recent study of GABA_A_-mediated inhibition in gerbil auditory cortex [23] and with previous studies that have demonstrated co-tuning of excitation and inhibition in AI [21] and suggest a role of auditory cortex in object representation rather than or in addition to feature extraction [24], [25].

Some of the units recorded did not show a response behavior as presented in Fig. 2: For example, in five pair recordings both units behaved like winners (cf. Results). This is not surprising, since one would not expect every single unit in the map to participate in the supposed ‘winner-take-all’-mechanism. At least some units in each BP-representation should respond to their preferred periodicity irrespective of concurrent sounds: In a cocktail-party situation, one has to be able to switch one's attention to a new sound source, for example if somebody is calling one's name. It is conceivable that some ’base’ activation for every sound source has to be maintained within the map to allow some top-down mechanism to switch the attention to another sound source.

The data from the competitive interaction experiments reported here support our hypothesis of a competitive “winner-take-all” algorithm that might be used by the auditory system to segregate a sound with a particular periodicity, such as an animal vocalization or a speech sound, from a mixture of simultaneously presented, concurrent sounds. By recording simultaneously from two recording locations, we were able to demonstrate that winner- and loser-type responses can be observed at the same time in a competitive interaction experiment. This observation is compatible with the idea of a direct suppressive interaction between units, although it is not yet direct evidence of such an interaction. However, we recently found such evidence with a stimulation paradigm as simple as a mere pure tone stimulation [29]. We could demonstrate that iontophoretic manipulation of a unit's response rate by either GABA (reduced response rate at the application site) or the GABA_A_-blocker gabazine (increased rate at the application site) leads to opposite effects on response rate at recording sites remote from the application site. Therefore it is also conceivable that the effects observed in our competitive interaction experiment indeed result from such direct suppressive interactions.

In addition to the known spatial cues [3], the mechanism described in this report may be used by the auditory system to segregate the speech of different speakers in cocktail-party-situations [26]. If this is the case, one would expect to find a neuronal correlate in AI of a switch in the focus of attention from one object to another [30], e.g., from one speaker to another in a cocktail-party situation. Interestingly, two of the ten winner-loser-pairs we recorded (as well as 5 individually recorded units) showed labile responses to AM-tone complexes which were suggestive of this type of behavior. In these cases, one unit was the ‘winner’ during measurements with the first set of two AM tone complexes, whereas the other unit was the ‘winner’ during measurements with the second set of stimuli. In addition, another five individually recorded units showed this type of ‘switching’ behavior. This suggests that different representations within the periodicity map may be ‘winners’ at different times. It should be emphasized that these experiments were performed on anesthetized animals, which might be an explanation for the low occurrence of this type of spontaneous ‘switching’ behavior. This phenomenon may be observed more frequently in awake animals. Indeed, it is conceivable that in the awake state there may never be a stable winner-loser-relationship between different representations in the periodicity map.

Using optical imaging of intrinsic signals, we could previously demonstrate the presence of a periodicity map with a circular topography in AI of the Mongolian gerbil [4]. The neuroanatomical data presented here show that intrinsic horizontal connections in AI have the appropriate topographical specificity and spatial extent to support the proposed competitive interaction mechanism within the cyclic periodicity map. Furthermore, these laterally projecting axons could indirectly mediate inhibitory interactions between different regions of the periodicity map given that a substantial fraction of their synaptic targets are inhibitory interneurons. We did not consider direct long-range inhibitory connections (for a review see Ref. [17], because in cortex they are reported to be extremely rare (e.g. 0.7–0.8% of callosal projecting neurons [31]). The pattern of interconnections necessary for a competitive interaction mechanism, where all representations within the parameter space are about equally interconnected with all other representations in the map (cf. Ref. [4]), is easily realized within a circular functional map, but is much more difficult to implement in a map with a linear functional gradient. Our data therefore may also explain the functional need for a circular topography of the periodicity map in AI.

From a functional point of view, it is not surprising that GABA_A_-mediated inhibition in AI does not seem to shape frequency receptive fields by some local contrast enhancing mechanism such as lateral inhibition [23]. The cells providing the input to AI are already tuned for pure-tone frequency by virtue of lateral inhibitory mechanisms operating at lower levels of the auditory pathway. There is no need for the auditory system to solve the same task repeatedly at multiple levels. Rather the auditory cortex seems to use the same network elements (GABAergic inhibition) to accomplish the demands of auditory processing which are more sophisticated than the extraction of simple acoustic features, e.g. object recognition and segregation. In line with this view is the observation that the auditory cortex is not required for simple tasks such as pure tone discrimination, but is crucial for the discrimination of more complex sounds that possess a virtual pitch percept [32]. For all these higher processing tasks, mechanisms that influence the whole cortical map via globally effective interactions rather than local contrast enhancing inhibition seem to be required.

## Materials and Methods

### Animal preparation

Animals were prepared under deep general (Halothane, Hoechst) and local anesthesia, according to procedures described in detail elsewhere [33]. Left auditory cortex was exposed by craniotomy, leaving the dura intact. For stereotaxic fixation during electrophysiological recordings a 2.5 cm long aluminum bar was fixed to the frontal bones with dental acrylic and served as a head anchor. Insect pins were inserted into the skull to improve the stability of the head anchor and to serve as reference electrodes. Animals were then transferred to an anechoic, sound-attenuated chamber. Anesthesia was maintained by ketamine (Ketavet, 50 mg/ml), xylazine (Rompun 2%) and isotonic sodium chloride solution (mixture 9∶1∶10) i.p. (0.06 ml/h). At the end of the recording session (after 20 to 24 h), animals were killed by an injection of T61 (Intervet) i.p. Experimental procedures were performed according to the federal regulations and were approved by the animal committee of the state of Saxony-Anhalt, Germany.

### Electrophysiological recordings

Anaesthetized animals were placed on a 37°C heating blanket to maintain body temperature with only the head fixed. All recordings were performed in a shielded, sound-attenuating chamber. Neural responses were recorded from primary auditory cortex (AI) with tungsten microelectrodes (TM3B10, 1 MΩ, WPI Inc., Sarasota, USA). Tracks were guided tangentially such that electrodes had a long track in the middle layers of AI. Unit activity was recorded using a multi-channel recording system (MAP ( = Multichannel Acquisition Processor), Plexon Inc.: amplification (20,000×), band-pass filter (250 Hz 2-pole low-cut filter and 8 kHz 6-pole high-cut filter), 40 kHz sampling at 12-bit resolution per recording channel). Spike waveforms of single units were separated online using a spike sorting algorithm (template matching: Sort Client software, Plexon Inc.), which allows a separation of 1 to 4 waveforms from multi-unit recordings. In the dataset presented in this study, we generally extracted only one spike from the multi-unit recording and the spike waveform was used to ensure the stability of the recording over the course of the experiments. Data from different spike clusters were stored separately for off-line analysis.

### Acoustic stimulation

Acoustic stimuli were delivered free field via an attenuator (PA4, Tucker Davies Inc.), an amplifier (STAX SRM-1/MK-2) and an electrostatic headphone (STAX SR lambda professional) which was mounted approximately 2 cm in front of the animal's head. The speaker's output was measured prior to an experiment using a ½-inch condenser microphone (Brüel & Kjaer 4190) placed at the position of the animal's head and facing the speaker using a measuring amplifier (Brüel & Kjaer 2610), and a signal analyzer (Brüel & Kjaer 2033). For frequencies between 0.3 and 20 kHz, the output of the speaker was found to be flat within ±5 dB and without distortion up to 90 dB SPL. Stimulus intensities higher than 90 dB SPL were not used.

To characterize basic neuronal response properties, pure tones (Fig. 1E) and AM tones (sinusoidally amplitude modulated pure tones) were produced by a computer-controlled multifunction generator (DD1, System 2, Tucker Davies Inc.). AM signals of 100% modulation depth were produced by adding three sine waves, viz. the carrier frequency fc and two sidebands with half the amplitude of fc (fc+modulation frequency (fm) and fc – fm). All components started at phase zero at stimulus onset. For the competitive interaction experiments, the best periodicity (BP; periodicity of the AM tone complex that elicited the highest response rate) for a given unit was determined from the responses to AM tones. Subsequently, the AM tone with the BP was presented simultaneously with a second AM tone complex with a different fc and varying fm (see Fig. 1F). In this case, both AM tone complexes had the same amplitude. All spectral components started at phase zero at stimulus onset. All stimuli were presented at a constant intensity of 65±5 dB SPL and had a duration of 200 ms with 5 ms rise and fall times. In some measurements where microiontophoretical application of BIC was performed (cf. below), stimuli were presented with 500 ms duration (cf. Fig. 4), as sometimes prolongations of neuronal responses have been reported during BIC-application (e.g. Refs. [13]–[16], [23]). Neuronal activity was also recorded during a 50 ms pre-stimulus and a 150 ms post-stimulus period. Stimuli were presented in random order with 15 repetitions of each stimulus, and were randomized separately for each repetition.

### Microiontophoresis

Three-barrel glass pipettes (3BBL W/FIL 1,2 MM, WPI Inc., Sarasota, USA), broken to a total tip diameter of 10–18 µm, were used for microiontophoresis. One barrel contained BIC (10 mM, Sigma (-)-bicuculline methiodide; Sigma), and the other two NaCl (3 M NaCl) for recording of neuronal activity and for current compensation. An Ionophor microiontophoresis system (Science Products) was used to generate and monitor ejection and retaining currents. To ensure that an adequate ejecting current was used, we performed control experiments with 4-barrel glass pipettes in which we first applied GABA iontophoretically with a current which was sufficient to inhibit a unit's response to its BF, and then ejected BIC with a current which antagonized the GABA-induced inhibition. These ejecting currents (20 to 40 nA) were then used to study the effect of BIC on responses to AM-tone complexes. The use of such low ejecting currents essentially excluded the possibility that iontophoresis of BIC would induce the well-documented side-effects of the drug which are not due to the blockade of GABA_A_-receptors (cf. Ref. [23]). Retaining currents ranged from −15 to −20 nA. For all cells, recordings were made before (control), during (BIC) and after (recovery) the application of BIC. For each of these conditions, responses to at least one set of pure tone stimuli were recorded. Measurements during the recovery condition were repeated until responses returned to pre-drug levels.

### Electrophysiological data analysis

Neuronal responses were visualized as rate functions and response plane histograms (cf. Figs. 2,4). Spontaneous activity was calculated from activity measured prior to stimulus onset and given in spikes/s (for rate functions) and spikes/bin for response plane histograms. The criterion for excitation was defined as spike activity significantly above spontaneous activity (spontaneous activity+3 standard deviations [SD], under the assumption that spike activity is Poisson-distributed). From the evoked responses (spike rate minus spontaneous activity) to AM tones we determined the best periodicity (BP; AM tone periodicity that evoked the highest discharge rate) and the evoked spike rate at the BP. The authors are aware of the fact that a number of stimulus properties co-vary with stimulus periodicity, like envelope rise time, pause duration, or spectral content. We nevertheless refer to best stimuli as ‘best periodicity’ considering that other stimulus features may influence response properties.

To describe tuning sharpness of responses to AM tones (in both single and double AM experiments) we defined a Q-value as BP divided by the bandwidth of the evoked response (that is, the response that is significantly above spontaneous activity in rate functions, as defined above).

A unit was defined as ‘winner’ if there was significant excitation visible in response plane histograms to all stimulus combinations in a competitive interaction experiment. Units where the response to at least one stimulus combination was below the criterion for significant excitation were defined as ‘losers’.

From rate functions filter types to AM tone complexes were defined as follows: The criterion for the classification of filter types was the number of crossings of the rate function with a line at 50% maximal response, and the location of these crossings relative to the BP. The filter function was defined as band-pass, if the rate function crossed the 50% criterion twice and started and ended below the criterion. The inverse case (2 crossings, start and end above criterion) was defined as band-suppression. The low-pass filter characteristic was defined by only one crossing, where the rate function started above and ended below the criterion. The inverse case (1 crossing, start below and end above criterion) was defined as high-pass. If there were more than two crossings of the 50% criterion, the filter characteristic of the rate function was defined as ‘complex’. Finally, if there was no crossing and the rate function was completely above the criterion, the response was defined as non-selective. Note that in the latter case no BP was determined.

### Neuroanatomy: Tracing and immunohistology

In order to access AI, gerbils (N = 10) were prepared as described above. Injections of 100 nl (N = 2), 20 nl (N = 2), or 5 nl (N = 6) of 5% biocytin (SIGMA-Aldrich Chemicals, Germany), dissolved in 0.05 M TRIS buffer (pH 7.6), were made by pressure (one injection per animal) over a period of two minutes using fine glass micropipettes (tip diameter 20 µm) and an oil hydraulic nanoliter delivery system (WPI, Germany) (Table 1). The injections were targeted at particular frequency regions of AI using best frequencies maps obtained in preceding electrophysiological experiments or using stereotaxic coordinates and features of the scull and cortical vasculature (for details see Ref. [28], [34]). Following the injections, the animals recovered and they were allowed to survive for 24 hours. They were then re-anaesthetized (0.5 mg ketamine/100 g body weight and 0.3 mg xylazine/100 g body weight, ip.) and perfused transcardially with 20 ml phosphate buffered saline (pH 7.4), followed by 200 ml of 4% paraformaldehyde and 0.1% glutaraldehyde in phosphate buffer (pH 7.4). The brains were removed, stored overnight in 4% paraformaldehyde at 4°C and then cut into 50 µm-thick sagittal sections using a vibratome (Leica Microsystems, Germany).

To visualize the transport of biocytin, sections were processed using the avidin-biotin-peroxidase reaction (ABC-kit, VECTOR Laboratories, USA) with diaminobenzidine as the chromogen. Then, consecutive sections were either not counterstained (for optimal visualization of traced connections), counterstained with methylgreen (to determine laminar and areal boundaries), or processed for parvalbumin (PV) (to identify putative inhibitory target cells of the traced connections). For PV staining, sections were first incubated in a solution of a PV-antibody (SIGMA-Aldrich, dilution of 1∶4000, 0.1% Triton) for 48 hours, then in a solution of a secondary antibody (biotinylated anti-mouse, SIGMA Aldrich, 1∶200) for two hours and visualized using the ABC method with α-chloronaphtol as the chromogen. After microscopic inspection (Leica Microsystems, Germany), digital photographs were taken (Finepix S2, Fuji, Japan) of the regions of interest (Fig. 6). For an appropriate illustration of small biocytin injections (5 nl), in two cases (G166, G171) the distribution of labeled boutons was reconstructed 3-dimensionally over several sections using Neurolucida software (MicroBrightField Europe) (Fig. 6B). Photomicrographs and illustrations were arranged using Adobe Photoshop software.
